# Plasma lipidomic signatures of spontaneous obese rhesus monkeys

**DOI:** 10.1186/s12944-018-0952-9

**Published:** 2019-01-08

**Authors:** Junlong Wang, Linqiang Zhang, Ruyue Xiao, Yunhai Li, Shasha Liao, Zhiguo Zhang, Wenhui Yang, Bin Liang

**Affiliations:** 1 Key Laboratory of Animal Models and Human Disease Mechanisms of the Chinese Academy of Sciences and Yunnan province, Chinese Academy of Sciences, Kunming Institute of Zoology, Kunming, 650223 China; 2 Center for Excellence in Animal Evolution and Genetics, Chinese Academy of Sciences, Kunming, 650223 China; 3 College of Pharmaceutical Sciences, Soochow University, Suzhou, 215123 China; 4 School of Life Sciences, Anhui University, Hefei, 230601 Anhui China; 5 Key Laboratory of Cardiovascular Disease of Yunnan Province, Department of Geriatrics, Yan’an Affiliated Hospital of Kunming Medical University, Kunming, China

**Keywords:** Obesity, Rhesus monkeys, Plasma lipidome, Palmitic acid (C16:0)

## Abstract

**Background:**

Obesity plays crucial roles in the pathogenesis of metabolic diseases such as hyperlipidemia, nonalcoholic fatty liver disease (NAFLD), and type 2 diabetes (T2D). The underlying mechanisms linking obesity to metabolic diseases are still less understandable.

**Methods:**

Previously, we screened a group of spontaneously obese rhesus monkeys. Here, we performed a plasma lipidomic analysis of normal and obese monkeys using gas chromatography/mass spectroscopy (GC/MS) and ultra-high performance liquid chromatography/mass spectroscopy (UPLC/MS).

**Results:**

In total, 143 lipid species were identified, quantified, and classified into free fatty acids (FFA), phosphatidylcholine (PC), phosphatidylethanolamine (PE), phosphatidylinositol (PI), phosphatidylserine (PS), phosphatidylglycerol (PG), lysophosphatidylcholine (LPC), lysophosphatidic acid (LPA), and sphingomyelin (SM). Data analysis showed that the obese monkeys had increased levels of fatty acids palmitoleic acid (C16:1) and arachidonic acid (C20:4), FFA especially palmitic acid (C16:0), as well as certain PC species and SM species. Surprisingly, the plasma level of LPA-C16:0 was approximately four-fold greater in the obese monkeys. Conversely, the levels of most PE species were obviously reduced in the obese monkeys.

**Conclusion:**

Collectively, our work suggests that lipids such as FFA C16:0 and 16:0-LPA may be potential candidates for the diagnosis and study of obesity-related diseases.

## Introduction

The increasing prevalence of obesity is becoming a medical and public health problem, since it is highly associated with metabolic abnormalities, including hyperlipidemia, nonalcoholic fatty liver disease (NAFLD), type 2 diabetes (T2D), and cardiovascular diseases [1, 2]. Numerous studies have shown the roles of obesity in the development of insulin resistance (IR) and T2D. It has been well recognized that obesity-caused dyslipidemia, which is characterized by an increase in plasma lipid content, especially triacylglycerols (TG) and free fatty acids (FFA), is a major factor contributing to the development of IR and T2D [2–4]. However, the precise mechanisms linking dyslipidemia to IR and T2D are still less understandable. Therefore, it is crucial and urgent to identify key factors involved in the pathogenesis of IR and T2D induced by obesity, especially at the early stage of disease progression.

Non-human primates (NHPs) such as the rhesus monkey (*Macaca mulatta*) and cynomolgus macaque (*Macaca fascicularis*) are uniquely appropriate for biomedical research due to their high similarity to human beings in genetics and physiology [5, 6]. Although obesity can be easily induced by high-calorie diets, many reports have convincingly demonstrated that spontaneous obesity is common in NHPs, and they displayed similar obesity-related physiologic changes, including increased abdominal fat, body mass index (BMI), as well as dyslipidemia IR and T2D, to those in humans [7–12]. Thus, NHPs have frequently been used as valuable research models for obesity and obesity-related metabolic diseases [7–12].

Lipidomics represents the pattern of global lipid species, and offers many promising novel lipid biomarkers and valuable information to elucidate the pathogenesis of common complex diseases, such as dyslipidemia and obesity [13, 14]. With the advent of novel detection and analysis technologies, it is becoming possible to perform comprehensive lipidomic analysis in plasma and tissues. Previously, we screened a certain number of spontaneously obese and diabetic rhesus monkeys [15]. Three individual female obese monkeys with a BMI of 40 kg/m^2^ or higher and age from 12 to 18 years were selected and their metabolic indexes subsequently monitored over 1 year, in which they displayed dyslipidemia, fatty liver, and IR, similar to human T2D at the early stage [15]. Nevertheless, lipid profiles of these obese rhesus monkey so far have not been reported. In the current study, we conducted a plasma lipidomic analysis of normal and spontaneously obese monkeys to define potential lipid biomarkers or disease factors for obesity and insulin resistance.

## Methods and materials

### Animals and collections of plasma samples

The criteria used to screen the spontaneously obese monkeys was described in our previous study, and obesity was defined as BMI of 40 kg/m^2^ or higher [15]. Three individual obese (OB) female monkeys and three normal (CK) female monkeys with similar ages (12–18 years) were identified as previously reported [15] and continuously used for this study. All monkeys were maintained under a 12-h light and dark cycle and had ad libitum access to water and food, which included 21.6% of calories as protein, 5.4% as fat, and 56.6% as carbohydrates.

A dosage of 20 mg/kg ketamine was used to euthanize the monkeys. The blood was collected into an ethylenediamine tetraacetic acid (EDTA)-containing tube from the femoral vein under fasting conditions, and then centrifuged under 3000 rpm for 5 min at room temperature. After centrifugation, the plasma was stored at − 80 °C for further study.

All animal procedures were carried out in strict accordance with the guidelines of the National Care and Use of Animals approved by the National Animal Research Authority (P.R. China) and the Institutional Animal Care and Use Committee (IACUC) of the Kunming Institute of Zoology of Chinese Academy of Sciences (KIZ, CAS). The nonhuman primate care and experimental procedures were approved by the Ethics Committee of Kunming Institute of Zoology and the Kunming Primate Research Center, Chinese Academy of Sciences (AAALAC accredited).

### Gas chromatography/mass spectroscopy (GC/MS) analysis of plasma fatty acid compositions

Plasma fatty acids were methylated following previously reported methods but with minor modifications [16–18]. In brief, 20 μL of internal standards in hexane containing 1 mg/mL of methyl heptadecanoate, 0.5 mg/mL of methyl tricosanoate, and 28 mg/mL of butylated hydroxytoluene (BHT) were added to a Pyrex tube followed by the addition of 50 μL plasma and 1 mL of methanol/hexane mixture (4:l, *v*/v). After cooling down the tubes in a self-made liquid nitrogen bath for 10 min, 100 μL of precooled acetyl chloride was added to the mixture and then flushed briefly with nitrogen gas. The tubes were then screw-capped and kept at room temperature in the dark for 24 h. The resultant mixture was cooled in an ice bath for 10 min followed by the gradual addition of 2.5 mL of 6% K_2_CO_3_ solution (with shaking) to neutralize. After standing for another 30 min, 200 μL of hexane was added to extract methylated fatty acids. The mixture was rested for 10 min, and the top layer was transferred into a glass sample vial. This extraction process was further repeated twice, and the combined supernatants were evaporated to dryness. The resultant residues were re-dissolved in 50 μL of hexane followed with GC- flame ionization detector (FID)/MS analysis.

Methylated fatty acids were measured on a Shimadzu GC/MS-QP2010Plus spectrometer (Shimadzu Scientific Instruments, USA) equipped with a MS with an electron impact (EI) ion source and an FID. An Agilent DB-225 capillary GC column (10 m, 0.1 mm ID, 0.1 μm film thickness) was employed with a sample injection volume of 1 μL and a splitter (1:60). Helium gas was used as carrier and makeup gas. The injection port and detector temperatures were both set at 230 °C. The column temperature was set to 55 °C for 1 min and then increased to 205 °C with a rate of 30 °C/min. The column temperature was then kept at 205 °C for 3 min and then increased to 230 °C (5 °C/min). The MS spectra were acquired with the EI voltage of 70 eV and the m/z range of 45 to 450. Methylated fatty acids were identified by comparing with a chromatogram from a mixture of 37 known standards and further confirmed with their mass spectral data. Each fatty acid was quantified with the FID data from its signal integrals and internal standards. The results were presented as μmol of fatty acids per liter of plasma.

### Ultra-high performance liquid chromatography/mass spectroscopy (UPLC/MS) analysis of plasma FFAs

An individual plasma sample (10 μL) was accurately transferred into an Eppendorf (EP) tube. Then, 10 μL of internal standard (200 ng/mL of heptadecanoic acid in methanol) was added to the tube, followed by the addition of 5 μL of (butylated hydroxytoluene) BHT, 100 μL of ultrapure water, 250 μL of methanol, and 12.5 μL of 1 N HCl. A biphasic solution was formed via the addition of 750 μL of isooctane. This solution was vortexed for 60 s, and the phases were separated by centrifugation at 3000 rpm for 60 s. The upper isooctane phase was collected, and the extraction process was repeated twice. The combined supernatants were evaporated to dryness. To the residue, we added 20 μL of ice-cold acetonitrile (MeCN)/N,N-dimethylformamide (DMF) (4:1, *v*/v) and 20 μL of ice-cold 640 mM [3-(dimethylamino)propyl]- ethylcarbodiimide (EDC) in ultrapure water. The tube was briefly mixed on a vortex mixer and placed on ice while the other samples were processed. To each tube, 10 μL of 20 mM N-hydroxybenzotriazole (HOBt) in MeCN/DMF (99:1, *v*/v) and 30 μL of 20 mM N-[4-(Aminomethyl)phenyl]pyridinium (AMPP) in MeCN were added, and placed in a 60 °C incubator for 30 min. The solution was filtered by a 0.22-μm membrane filter before UPLC-QQQ-MS/MS analysis.

Samples were analyzed by liquid chromatography (Agilent 1290, San Jose, CA, USA) coupled to electrospray ionization on a triple quadrupole mass spectrometer (Agilent 6460, San Jose, CA, USA). Chromatographic separation was achieved on an ZORBAX Eclipse Plus C18 (2.1 × 100 mm, 1.8 μm particles; Agilent) column using a flow rate of 0.5 mL/min at 40 °C during a 13-min gradient (0–12 min from 30% B to 95% B, 12–13 min 95% B), while using the solvents A, 100% water containing 0.1% formic acid, and B, 100% acetonitrile containing 0.1% formic acid. Electrospray ionization was performed in the positive ion mode using N2 at a pressure of 20 psi for the nebulizer with a flow of 10 L/min and a temperature of 350 °C. The sheath gas temperature was 350 °C with a flow rate of 10 L/min. The capillary was set at 4000 V.

To detect the individual fatty acids, (multiple reaction monitoring) MRM in positive ion mode was performed with individually optimized fragmentor voltage and collision energies (Optimizer application, Mass-Hunter, Agilent). MRM transitions were achieved by flow injection of pure standards and the optimizer application and were compared to the literature when available for certain compounds.

### UPLC-MS analysis of plasma phospholipids and sphingolipids

An individual plasma sample (50 μL) was accurately transferred into an EP tube. Next, 10 μL of internal standard (10 μg/mL) in methanol was added to the tube, followed by 10 μL of BHT in methanol and 1 mL of chloroform (MeCl)/methanol (MeOH) (1:1, *v*/v). The solution was left at room temperature for 2 min and then added to 0.45 mL of ultrapure water and centrifuged at 10,000 rpm for 10 min at 4 °C. The lower phase containing the lipids was collected, and the extraction process was repeated once with the upper layer. The combined lower solutions were evaporated to dryness. The resultant residues were re-dissolved in 80 μL of MeCl/MeOH (1:1, *v*/v) followed by filtering with a 0.22-μm membrane filter before UPLC-QQQ-MS/MS analysis.

Samples were analyzed by liquid chromatography (Agilent 1290, San Jose, CA, USA) coupled to electrospray ionization on a triple quadrupole mass spectrometer (Agilent 6460, San Jose, CA, USA). Briefly, 2 μL of phospholipids extract were injected into an analytical column, ZORBAX Eclipse Plus C18 (2.1 × 100 mm, 1.8 μm particles; Agilent). A binary isocratic elution with 98%B was applied with water for solvent A and methanol (0.01% formic acid, 5 mM ammonium acetate) for solvent B. The column was held at 50 °C, and the separation was allowed at a flow rate of 0.5 mL/min. The fragmentor, collision energy, and other experimental conditions were adjusted for each phospholipid.

The product ion mode was used to identify the fatty acid composition of phospholipid species. To detect the individual phospholipid, MRM in positive/negative ion modes was performed with individually optimized fragmentor voltage and collision energies (Optimizer application, Mass-Hunter, Agilent). MRM transitions were achieved by flow injection of pure standards and the optimizer application and were compared to the literature when available for certain compounds.

### Statistical analysis

Data were presented as mean ± standard error of the mean (SEM), unless specially indicated. Statistical differences were analyzed by t test using SPSS 10.0 (IBM SPSS statistics, Armonk, NY, USA), and a value of *p*<0.05 was regarded as significant. All figures were made using GraphPad Prism 7.0 (Graphpad Software, La Jolla, CA, USA).

## Results

### Obese monkeys showed increased levels of FFA

Our previous report identified a group of spontaneously obese rhesus monkeys with significantly increased boy weight, BMI, and TG content in serum and liver, as well as mild insulin resistance [15] . To define potential lipid biomarkers or disease factors involved in obesity and insulin resistance, the blood samples of three individual obese (OB) and normal (CK) monkeys, which were used for liver proteome analysis previously [15], were also simultaneously collected for lipidome analysis.

**Fig. 1:**
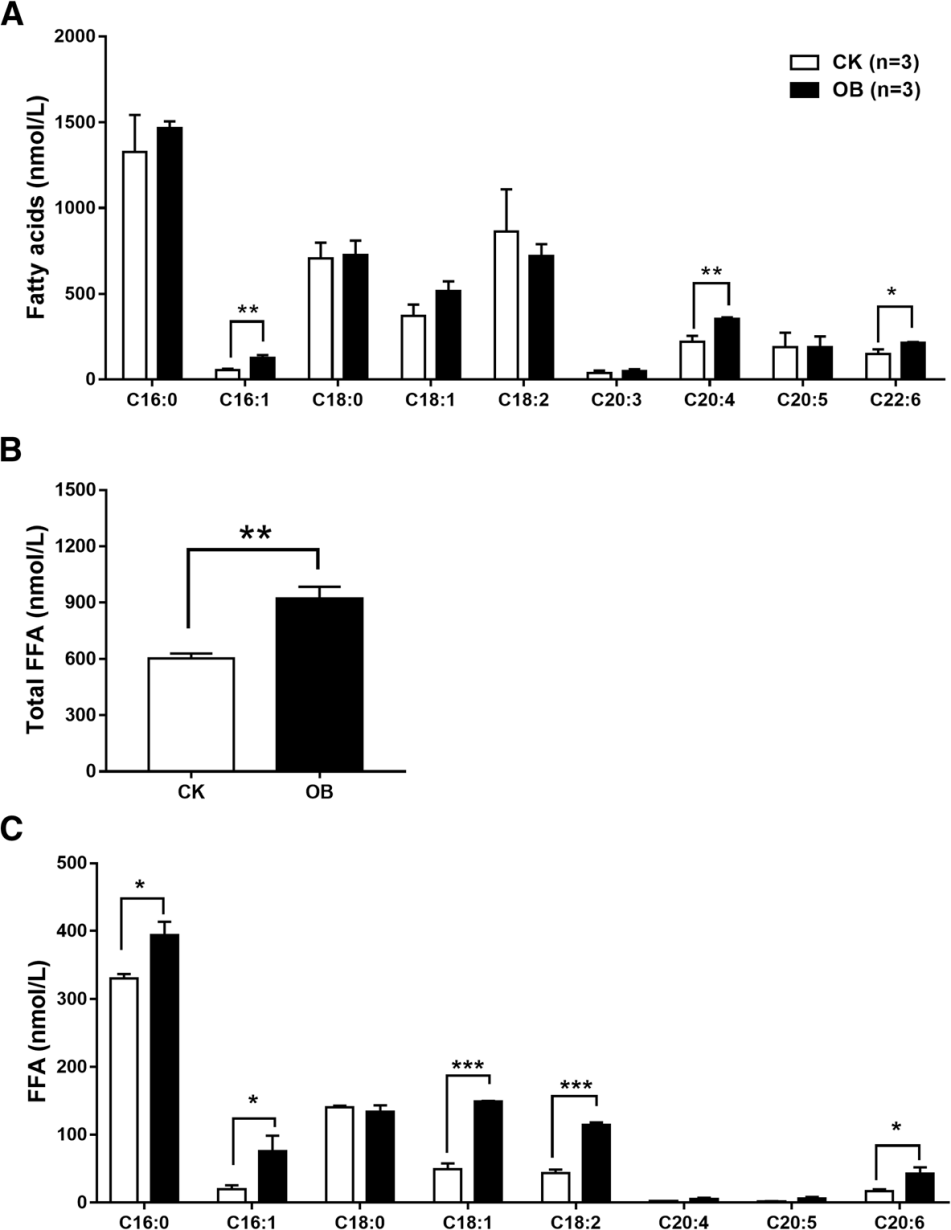
Levels of plasma fatty acid compositions and free fatty acids in obese monkeys (OB, *n* = 3) and normal monkeys (CK, *n* = 3). Levels of fatty acid composition (**a**), total free fatty acids (**b**), and free fatty acids (FFAs) (**c**). Data are presented as mean ± standard error of the mean (SEM). Significant difference between CK and OB monkeys: *P<0.05, **P<0.01

GC/MS was performed to determine fatty acid species. Although the levels of total plasma fatty acids had no difference (data not shown), the levels of both palmitoleic acid (C16:1) and arachidonic acid (C20:4) were significantly increased in the obese monkeys compared with the normal monkeys (Fig. 1a). Many studies reported that increased levels of FFAs are implicated in the pathogenesis of insulin resistance [19, 20]. We therefore examined plasma FFA levels. Indeed, the level of total FFA was significantly increased in the obese monkeys compared with the normal monkeys (Fig. 1b). Consistent with human results [21], palmitic acid (C16:0) was the major constituent fatty acid among eight FFA species examined in monkey plasma (Fig. 1c). Of note, excepting C20:4 and C20:5, for which their levels were too low, the levels of five fatty acids including C16:0, C16:1, C18:1, C18:2, and C20:6 were increased in the obese monkeys compared with the normal monkeys (Fig. 1c). Taken together, these results demonstrate that obese monkeys also display an increased FFA level, which is consistent with human subjects and the rodent model of obesity.

### Profiles of plasma phospholipids and sphingolipids

**Fig. 2:**
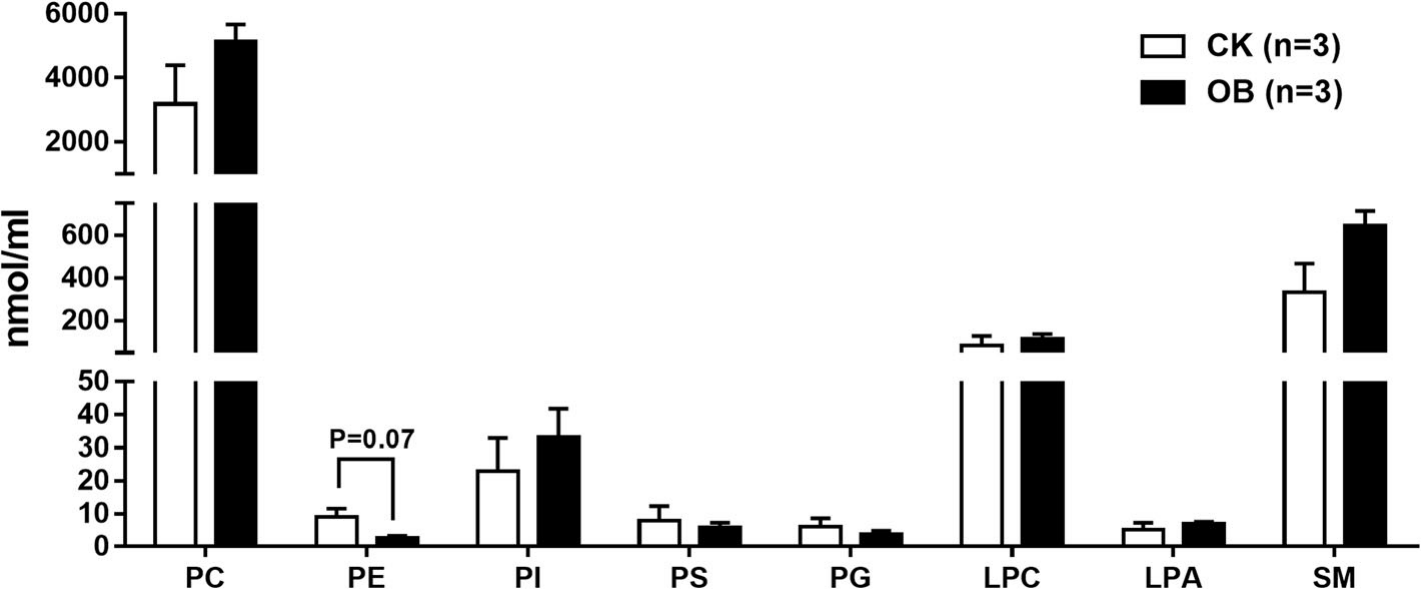
Levels of plasma phospholipids and sphingolipids in obese monkeys (OB, *n* = 3) and normal monkeys (CK, *n* = 3). Levels of total phosphatidylcholine (PC), phosphatidylethanolamine (PE), phosphatidylinositol (PI), phosphatidylserine (PS), phosphatidylglycerol (PG), lysophosphatidylcholine (LPC), lysophosphatidic acid (LPA), and sphingomyelin (SM). Data are presented as mean ± standard error of the mean (SEM)

A UPLC/MS-based approach was carried out to profile plasma phospholipids (PL) and sphingolipids (SM) of normal and obese monkeys. A total of 129 lipid species were identified, quantified, and classified from phosphatidylcholine (PC), phosphatidylethanolamine (PE), phosphatidylinositol (PI), phosphatidylserine (PS), phosphatidylglycerol (PG), lysophosphatidylcholine (LPC), lysophosphatidic acid (LPA), and SM. PC is the most abundant lipid class, followed by SM, LPC, and PI, among eight classes of lipid species (Fig. 2). In summary, the levels of plasma PC, SM, and LPA were somewhat increased, whereas the level of plasma PE was slightly decreased (*P* = 0.07) in the obese monkeys compared with the normal monkeys (Fig. 2).

### Obese monkeys showed increased levels of phosphatidylcholine (PC) species

**Fig. 3:**
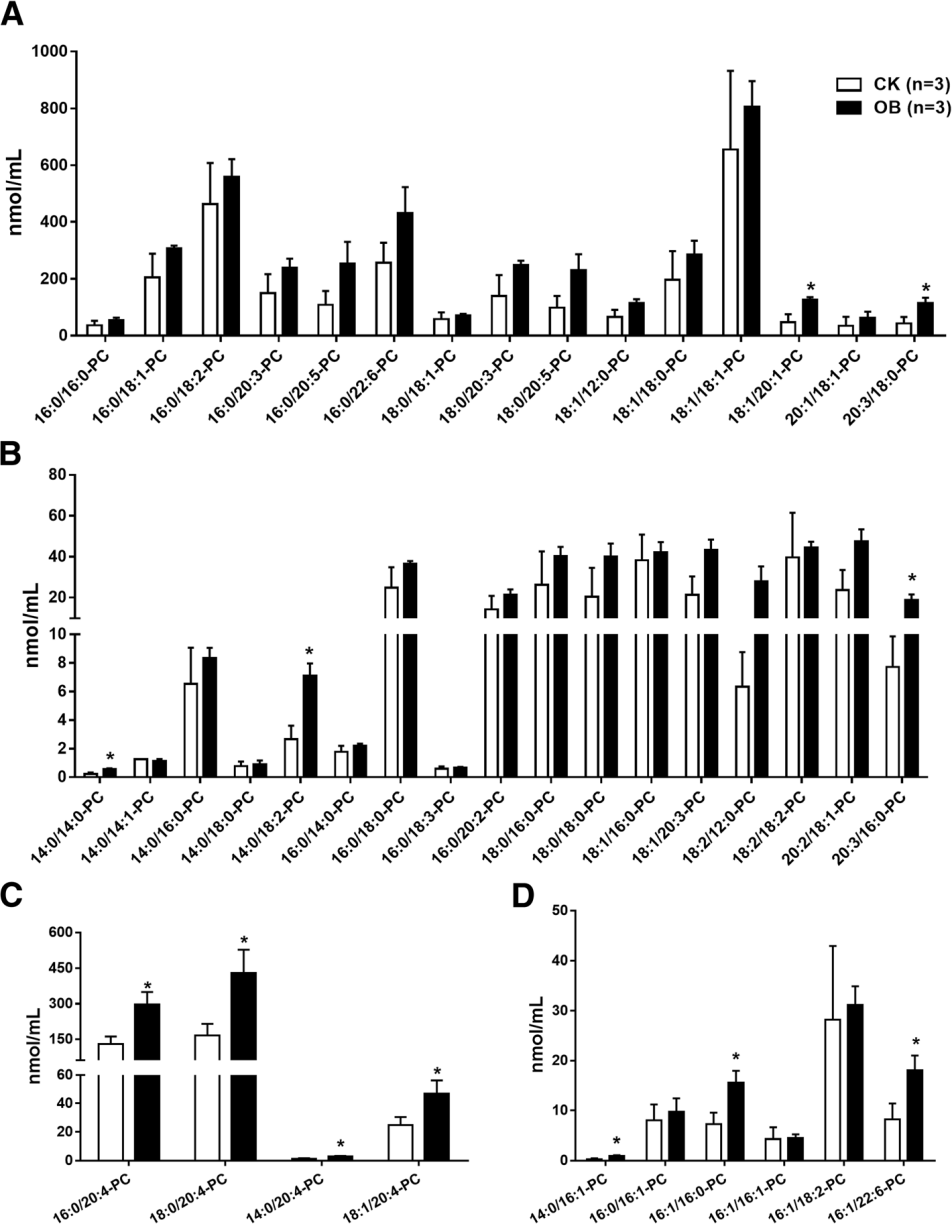
Levels of plasma phosphatidylcholines (PC) species in obese monkeys (OB, n = 3) and normal monkeys (CK, n = 3). **a** Levels of PC species with an average concentration > 50 nmol/mL. **b** Levels of PC species with an average concentration < 50 nmol/mL. **c** Levels of PC species containing fatty acid C20:4. **d** Levels of PC species containing fatty acid C16:1. Data are presented as mean ± standard error of the mean (SEM). Significant difference between CK and OB monkeys: *P<0.05

In total, 42 PC species were detected and quantified, of which 17 PC species showed an average concentration greater than 50 nmol/mL (Fig. 3a and c), but 25 showed an average concentration less than 50 mmol/mL (Fig. 3b and d). In addition, the concentration of all eight PC species with C14:0 fatty acid was below 10 nmol/mL (Fig. 3b). Generally, the levels of most PC species were mildly increased in the obese monkeys compared with the normal monkeys (Fig. 3a and b). As mentioned, the levels of fatty acids C16:1 and C20:4 were significantly increased in the obese monkeys (Fig. 1a). Consistently, the levels of all four PC species with C20:4 (Fig. 3c), but only half of PC species with C16:1 (Fig. 3d), were significantly increased in the obese monkeys compared with the normal monkeys. These results suggest that the creased levels of fatty acid C20:4 may derive from PC, but not FFAs.

### Obese monkeys showed decreased levels of phosphatidylethanolamine (PE) species

**Fig. 4:**
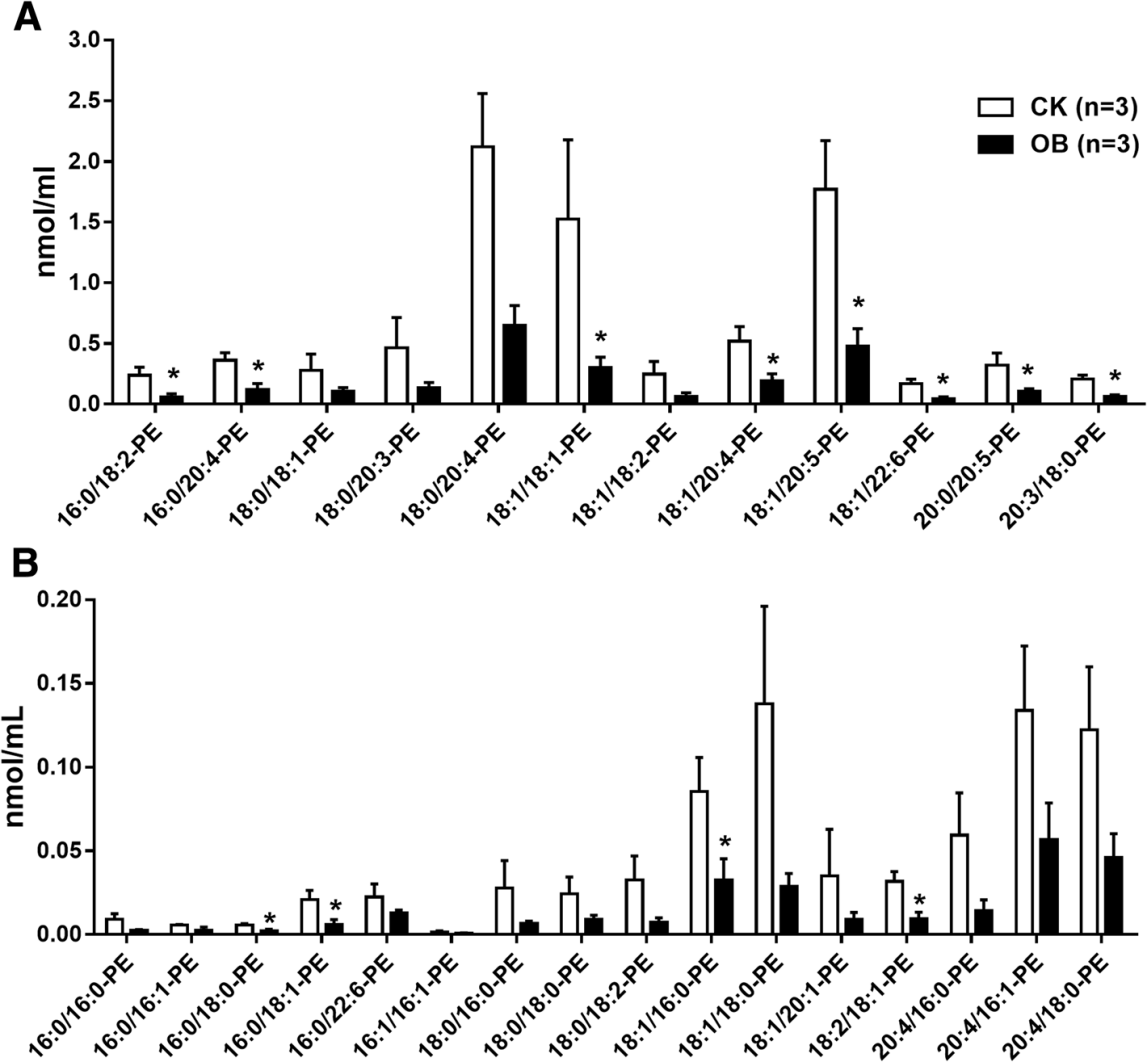
Levels of plasma phosphatidylethanolamine (PE) species in obese monkeys (OB, *n* = 3) and normal monkeys (CK, *n* = 3). Levels of high abundant PE species (**a**) and low abundant PE species (**b**). Data are presented as mean ± standard error of the mean (SEM). Significant difference between CK and OB monkeys: *P<0.05

As mentioned previously, contrary to PC, the total level of PE, although it was relatively very low, was mildly decreased in the plasma of obese monkeys (Fig. 2). A total of 28 PE species were detected and quantified (Fig. 4a and b). Consistently, the levels of nearly all PE individual species were obviously decreased in the obese monkeys compared with the normal monkeys (Fig. 4a and b). In particular, the levels of 12 PE species were significantly decreased (Fig. 4a and b). These results may suggest a reverse association between plasma PE level and obesity.

### Obese monkeys showed increased levels of sphingomyelin (SM) species

**Fig. 5:**
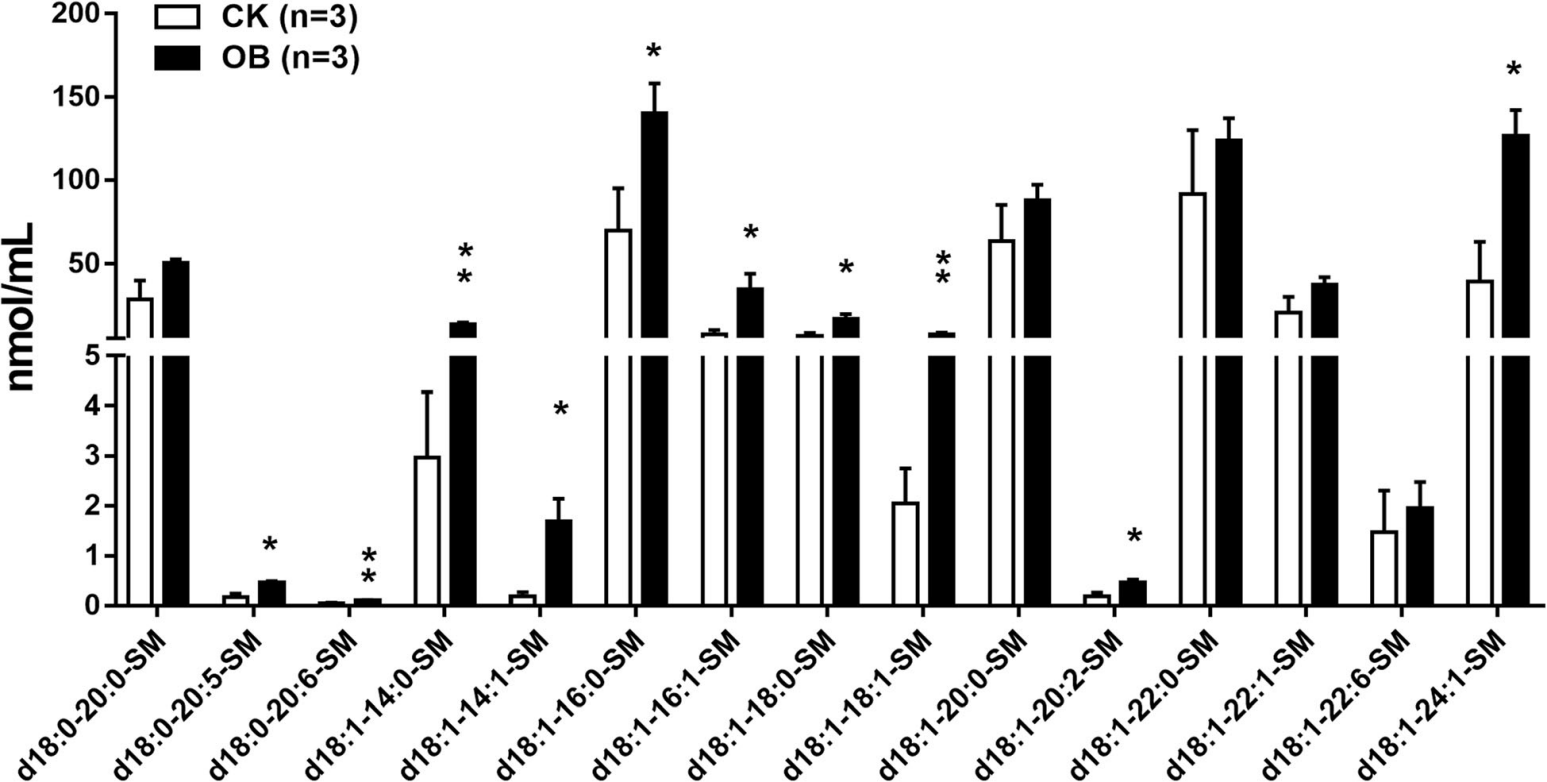
Levels of plasma sphingomyelins (SM) species in obese monkeys (OB, *n* = 3) and normal monkeys (CK, *n* = 3). Data are presented as mean ± standard error of the mean (SEM). Significant difference between CK and OB monkeys: *P<0.05, **P<0.01

Sphingomyelins (SM) are the major sphingolipids in the peripheral blood. Recent studies showed that increased plasma SM levels are associated with the development of obesity-related diseases such as type 2 diabetes and NAFLD [22, 23]. Similarly, 15 SM species were identified and quantified, of which 10 species levels were significantly increased in the obese monkeys compared with the normal monkeys (Fig. 5). Interestingly, all SM species contained a C18:1 fatty acid. Conversely, none of the SM species contained a C20:4 fatty acid. These results suggest that increased levels of SM species may also be associated with hyperlipidemia, obesity, and IR in rhesus monkeys.

### Profiles of lysophosphatidylcholines (LPC), lysophosphatidic acids (LPA), phosphatidylinositols (PI), and phosphatidylserines (PS) species

**Fig. 6:**
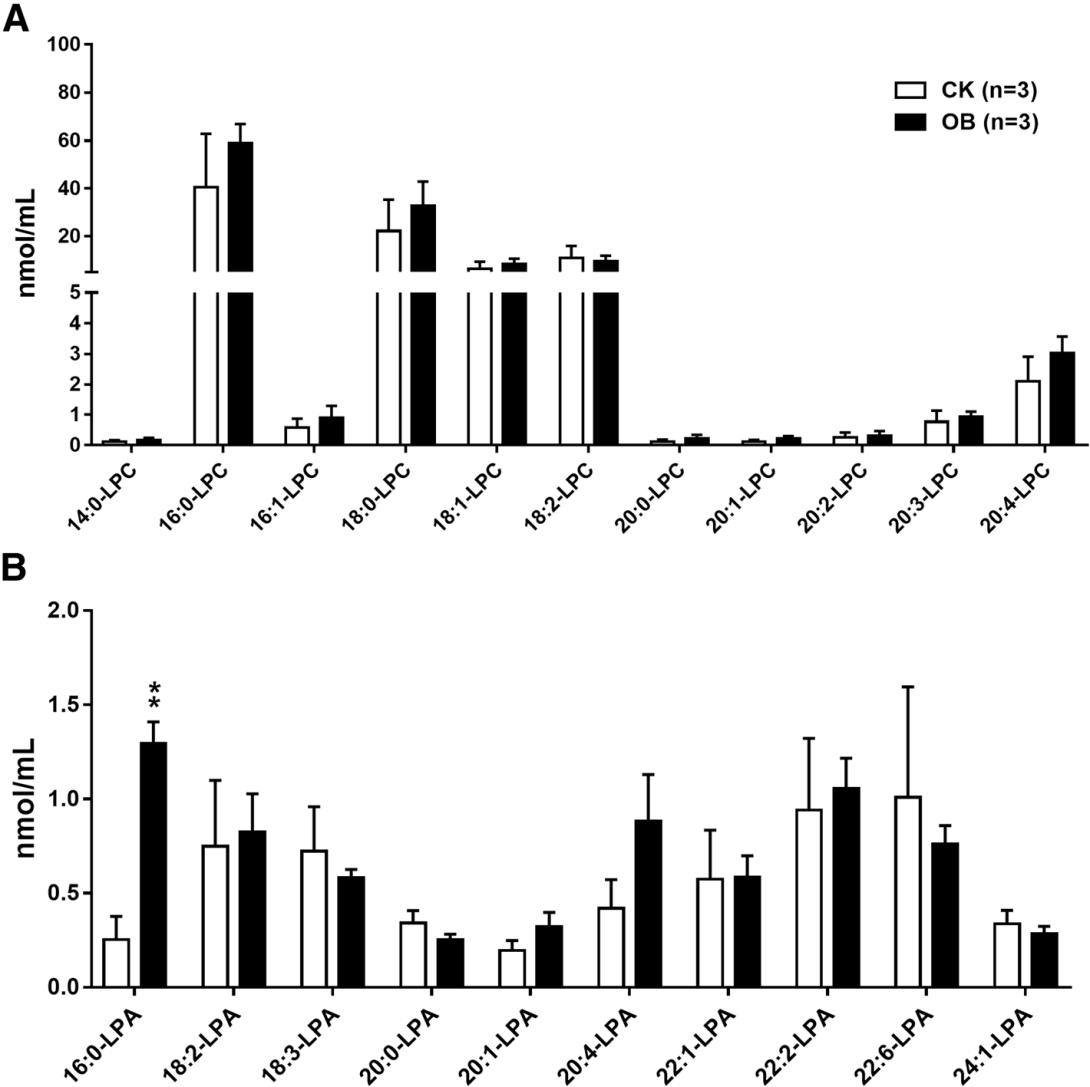
Levels of plasma lysophosphatidylcholines (LPC) and lysophosphatidic acids (LPA) species in obese monkeys (OB, *n* = 3) and normal monkeys (CK, *n* = 3). Levels of lysophosphatidylcholines (LPC) (**a**) and lysophosphatidic acids (LPA) species (**b**). Data are presented as mean ± standard error of the mean (SEM). Significant difference between CK and OB monkeys: **P<0.01

**Fig. 7:**
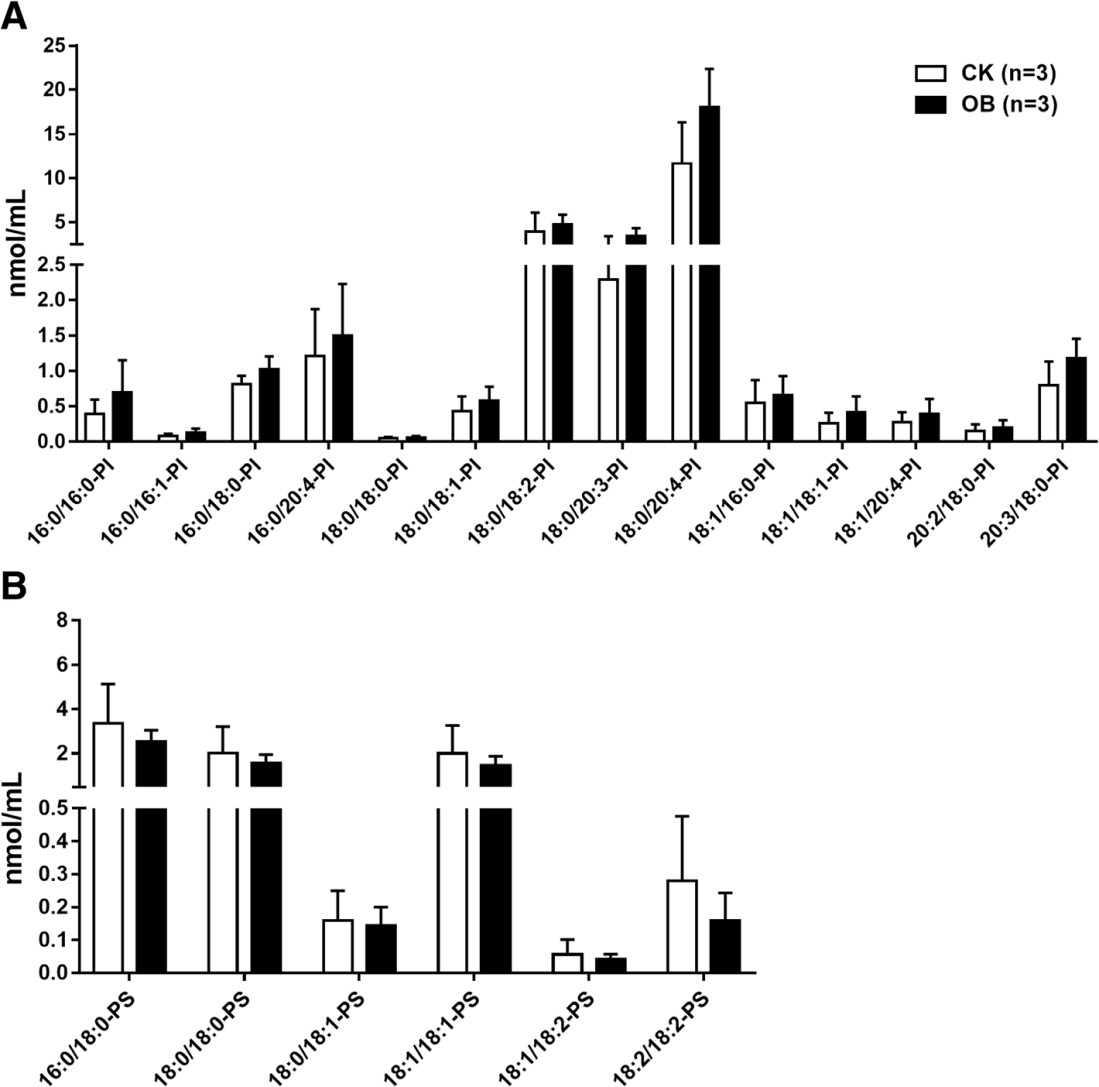
Levels of plasma phosphatidylinositols (PI) and phosphatidylserines (PS) species in obese monkeys (OB, *n* = 3) and normal monkeys (CK, *n* = 3). Data are presented as mean ± standard error of the mean (SEM)

In addition, 11 LPC species (Fig. 6a), 10 LPA species (Fig. 6b), 14 PI species (Fig. 7a), and 6 PS species (Fig. 7b) were also identified. Generally, the levels of all LPC, PI, and PS species were no different between the obese monkeys and the normal monkeys. Consistent with humans [21], the concentrations of LPA species were very low in the plasma of rhesus monkeys. Interestingly, the plasma level of LPA C16:0 was approximately four-fold in the obese monkeys of that in the normal monkeys (Fig. 6b), suggesting that LPA C16:0 level may be associated with hyperlipidemia, obesity, and IR.

## Discussion

Obesity-related diseases often display distinct changes in global blood lipid profiles, such as triacylglycerols and phospholipids. It is well recognized that impaired lipid metabolism plays an important role in the development of NAFLD, IR, and T2D. The plasma levels of total cholesterol (TC), total triacylglycerols (TG), and low-density lipoprotein cholesterol (LDL-c) are predominantly employed as clinical diagnosis makers for these metabolic diseases. Our previous study screened a certain number of spontaneously obese rhesus monkeys that displayed mild IR and increased TG accumulation in both plasma and liver [15].

To date, several studies have examined the plasma lipidomics in both rodent models of obesity and human obesity [24–27]. Plasma lipidomic studies on obese mice showed that total PC, TG, LPA [28, 29], and FFAs levels were increased, whereas the levels of most LPCs, such as LPC (14:0), LPC (15:0), and LPC (16:0), were decreased [14, 24, 26, 30]. Similarly, the levels of diacylglycerol (DAG), TG, FFA, and most of PL classes, such as PC, PI, and PS, were increased in obese and NAFLD patient subjects [31–34].

In the present study, our plasma lipidomic analysis identified 143 lipid species including 9 FAs, 8 FFAs, 42 PCs, 28 PEs, 14 PIs, 6 PSs, 11 LPCs, and 10 LPAs, as well as 15 SMs in rhesus monkeys. Furthermore, compared with the normal monkeys, the obese monkeys showed increased levels of total FFA (Fig. 1b), as well as most of the PC species (Fig. 2) and SM species (Fig. 5); however, the levels of the majority of PE species were decreased. To the best of our knowledge, this may be the first plasma lipidome of rhesus monkeys.

Analysis of plasma fatty acid compositions showed that, of all nine detected fatty acids, only the levels of two fatty acids, palmitoleic acid (C16:1) and arachidonic acid (C20:4), were significantly increased in the obese monkeys (Fig. 1a), in accordance with a report of serum metabolomics that obese patients had increased level of serum palmitoleic acid, which could be used to predict the future development of metabolic syndrome (MS) [34]. Furthermore, the increased levels of fatty acids C16:1 and C20:4 were actually found in FFA (Fig. 1c) and PC (Fig. 3c), respectively. In humans, the most abundant fatty acid in plasma is the saturated fatty acid C16:0 [21], which may promote liver injury and insulin resistance [35–37]. Likewise, this phenomenon was also present in rhesus monkeys, in which the C16:0 fatty acid was not only the highest abundant fatty acid (Fig. 1a), but also its level was notably increased in FFA (Fig. 1c) in the obese monkeys (Fig. 1c). Meanwhile, the level of C16:0-LPA was also markedly increased in the obese monkeys (Fig. 6b). An increased plasma level of FFA is a signature of obesity and insulin resistance [19, 20]. Therefore, plasma total FFA as well as FFAs C16:0 and C16:1 are probably valuable diagnostic lipid indicators for obesity and IR.

Of note, consistent with plasma lipidome studies in humans and cynomolgus monkeys [21], PC was the most abundant phospholipid in rhesus monkeys as well (Fig. 2). Several studies found an increased PC level in high fat induced obese mice and rats [38, 39]. Likewise, although the level of total PC only showed an increased trend, the levels of a number of PC species, especially C20:4 PC species, were significantly increased in obese monkeys (Fig. 3). On the contrary, the levels of most PE species were significantly lower in obese monkeys. A previous report also showed decreased PE levels in NAFLD patients [33]. However, several studies found that obesity increased the content of specific PE species in plasma, such as PE 38:4 and PE 16:0/22:4 [31, 39]. Therefore, the plasma PE level in obesity-related diseases needs to be further investigated.

LPC is an important signaling molecule and plays a variety of biological functions, such as inflammation, cell proliferation, and insulin resistance [40–42]. Many studies have showed a general decrease of circulating LPC species, especially LPC 16:0, in high-fat induced rodent models and obese humans [14]. However, neither the level of total LPC (Fig. 2), nor the levels of LPC species (Fig. 6a), were altered in the obese monkeys compared with the normal monkeys. Surprisingly, we found that the level of LPA 16:0 was dramatically increased in the plasma of obese monkeys (Fig. 6b). Whether LPA 16:0 can be considered to be a potential biomarker for obesity-disease diagnosis needs to be further characterized in the future.

A great number of studies have reported the obesity-associated alteration in SM species. Elevated levels of total SM and individual SM species were found in both obese patients and high-fat induced obese rodent models. Similarly, plasma lipidome showed that the levels of most SM species were significantly increased in obese monkeys. Therefore, these lines of evidences including our current study consistently demonstrate that plasma SM levels may positively correlate with obesity and obesity-related disease.

## Conclusion

In summary, many of our findings in rhesus monkeys are consistent with reports from rodents and humans. Meanwhile, we also found a novel lipid species 16:0-LPA with an increased level in obese monkeys. These lipid species, especially FFA C16:0 and 16:0-LPA, with alteration may be candidates for the diagnosis and study of obesity-related diseases.
